# Mood Stabilizers in Psychiatric Disorders and Mechanisms Learnt from In Vitro Model Systems

**DOI:** 10.3390/ijms22179315

**Published:** 2021-08-27

**Authors:** Ritu Nayak, Idan Rosh, Irina Kustanovich, Shani Stern

**Affiliations:** Sagol Department of Neurobiology, University of Haifa, Haifa 3498838, Israel; rnayak@campus.haifa.ac.il (R.N.); irosh@campus.haifa.ac.il (I.R.); kustanz1@gmail.com (I.K.)

**Keywords:** schizophrenia, bipolar disorder, lithium, valproic acid, induced pluripotent stem cells

## Abstract

Bipolar disorder (BD) and schizophrenia are psychiatric disorders that manifest unusual mental, behavioral, and emotional patterns leading to suffering and disability. These disorders span heterogeneous conditions with variable heredity and elusive pathophysiology. Mood stabilizers such as lithium and valproic acid (VPA) have been shown to be effective in BD and, to some extent in schizophrenia. This review highlights the efficacy of lithium and VPA treatment in several randomized, controlled human trials conducted in patients suffering from BD and schizophrenia. Furthermore, we also address the importance of using induced pluripotent stem cells (iPSCs) as a disease model for mirroring the disease’s phenotypes. In BD, iPSC-derived neurons enabled finding an endophenotype of hyperexcitability with increased hyperpolarizations. Some of the disease phenotypes were significantly alleviated by lithium treatment. VPA studies have also reported rescuing the Wnt/β-catenin pathway and reducing activity. Another significant contribution of iPSC models can be attributed to studying the molecular etiologies of schizophrenia such as abnormal differentiation of patient-derived neural stem cells, decreased neuronal connectivity and neurite number, impaired synaptic function, and altered gene expression patterns. Overall, despite significant advances using these novel models, much more work remains to fully understand the mechanisms by which these disorders affect the patients’ brains.

## 1. Introduction

The global incidence of psychiatric disorders has increased over the past decades, placing a high socio-economic burden. However, suitable medications remain an unmet requirement [1]. Psychiatric disorders are a group of mental illnesses that, according to the World Health Organization (WHO), can be characterized as a combination of abnormal thoughts, perceptions, emotions, behavior, and relationships with others [2]. Psychiatric disorders include depression, BD, schizophrenia, and other psychoses [3,4]. In 2016, more than one billion people worldwide were affected by mental disorders, making up around 16% of the world’s population [5]. Both women and men are affected to a similar degree; however, women are more likely than men to develop BD later in life [6]. BD type I occurs more in men than in women [7,8], whereas BD type II/hypomania, rapid cycling, and mixed episodes are more prevalent in women than in men [9]. In schizophrenia, an extensive literature review suggests a higher rate in men and differences in symptoms between genders [10].

So far, several therapies have been researched dynamically for psychiatric disorders highlighting the importance of developing strategies that prevent subsequent relapse and recurrence of diseases. Psychiatric disorders are treated primarily through psychotherapy [11], deep brain stimulation [12], and medications. However, with psychotherapy and brain stimulation, many patients report incomplete symptoms resolution and relapse of disease. Therefore, the search for better treatments is still ongoing, and the focus remains mainly on the psychoactive effects of medications due to their disease-specific action [13]. One major line of treatment for psychiatric disorders, especially for BD, is mood stabilizers, along with other antimanic and anti-depressant drugs [14]. The mood stabilizers lithium and the anticonvulsant VPA are often used to treat BD [15]. However, many emerging studies indicate their ability to treat other neuropsychiatric, neurologic, and neurodegenerative disorders [15]. Furthermore, lithium is sometimes used for the treatment of psychotic symptoms in schizophrenia [16]. On the other hand, the use of VPA has been increasing in schizophrenia patients in recent years [17].

The complex dynamic system and the inaccessibility for experimental manipulation have created a challenge for researchers to study the brain. Earlier, researchers utilized animal models to understand brain development and function. Nevertheless, the neuroanatomical differences between humans and animals make animal models of brain diseases inaccurate. Thus, there is a lack of fundamental molecular data due to failure in recapitulating the disease phenotypes. Additionally, the genetic complexity and heterogeneity of psychiatric disorders make it difficult to create a good animal model [18]. Researchers required an in-vitro model system to help elucidate human brain pathophysiology. Hence, human cell culture models have recently been generated. Human neurons derived from induced pluripotent stem cells (iPSCs) are great candidates for modeling these disorders. The cell culture model system bestows a promising tool to create new disease models. Investigators have been able to use many different human-derived cell culture brain models such as neurospheres [19], neurons [20], astrocytes [21], microglia [22], oligodendrocytes [23], and the recent progress in developing 3D brain organoids and specific brain organoids such as cortical [24], midbrain [25], striatal [26], and hippocampal [27] organoids from inducible pluripotent stem cells [28]. 

In this review, we will focus on two psychiatric disorders, BD and schizophrenia, and on two mood stabilizers, lithium and VPA, highlighting their reported mechanism of action on various models of psychiatric disorders.

## 2. Psychiatric Disorders

### 2.1. Bipolar Disorder 

BD is a repetitive chronic disorder characterized by periodic fluctuation in temperament and vigor, causing a progressive functional and cognitive impairment that affects more than 1% of the world’s population [29]. The disorder usually consists of both manic and depressive episodes segregated by periods of normal mood [30]. BD affects about 45 million people worldwide [2] and has a typical age onset in early adulthood between 25 and 50 years of age [31]. To date, its biological basis is still poorly understood, and its treatment is unsatisfactory. 

This disorder was previously named manic-depressive disorder and was first described in 1851 by Jean Pierre Falret [32]. Through long-term observations, Falret developed the entity “Folie circulaire”, which means circular madness. Later in 1854, Jules Baillarger called it “Folie a double-form.” This idea was further supported by Emil Kraepelin, who later separated manic-depressive insanity from dementia praecox, paranoia, and paraphrenia [33]. 

BD is characterized by episodes of mania leading to overactivity, extreme energy, more distractions than usual, intense senses, sleep deprivation, jabbering, or extreme irritability, delusions, and hallucinations [34]. There are two major types of BD; bipolar I disorder includes the existence of at least one episode of mania and usually with at least one depressive episode, while bipolar II consists of at least one episode of hypomania and one episode of major depression (MD) [35]. Mania and hypomania are separated based on the extremity of the symptoms such as functional disability and psychotic behavior that ultimately leads to hospitalization in full-pledged mania. In contrast, in hypomania, the patients’ behavior is less extreme [36]. The average age of onset for bipolar I disorder is 18.2 years and 20.3 years for bipolar II disorder [37]. BD patients may belong to different classes based on their severity, duration, and symptoms [30].

#### 2.1.1. Neuroimaging Studies Associated with BD

The primary symptoms of BD such as psychosis, cognitive deficits, and hyperactivity-related disturbances suggest the involvement of neuroanatomic abnormalities that may differentiate patients with BD from healthy subjects. Advancements in functional neuroimaging studies have identified various anomalies associated with BD pathophysiology. Hibar et al., in 2018, addressed the broadest research to date on cortical gray matter thickness and surface area from magnetic resonance imaging (MRI) scans of 6503 BD and 2582 healthy individuals. Their results displayed reduced cortical thickness in the frontal, temporal, and parietal regions in BD adults, while young adolescent BD patients showed cortical thinning in the supramarginal gyrus and insula. Furthermore, the study included gender-specific diagnoses that showed broader cortical layers in adult female BD compared to the male patients [38]. 

The anterior cingulate cortex is another region that became noticeable in 1997 by Drevets et al., who used positron emission tomographic (PET) images and glucose metabolism rate to report abnormally reduced activity in the prefrontal cortex located ventral toward the genu of the corpus callosum [39]. Subsequently, Bouras et al., in 2001, analyzed neuronal density and size using the nissl staining technique by utilizing post mortem samples from 21 sporadic BD and 55 control brain sections. Using immunocytochemistry and immunodot methods, they showed reduced MAP2 protein expression in BD patients. Thus, BD patients show decreased laminar thickness and neuronal density in layers III, V, and VI of the subgenual part [40].

The prefrontal cortex (PFC) is one of the most widely studied brain regions in BD. In 2000, Guidotti et al. reported their neuropathological studies of the PFC in BD. Superior PFC gyrus of Brodmann area 9 specimens were collected from 60 subjects, out of which 15 were control brains, 15 were post-mortem brains from BD patients, 15 were brains of schizophrenia patients, and 15 brains were collected from major depression patients. RT-PCR and western blot analyses were conducted to quantify Reelin and GAD67 protein expression. Immunohistochemistry was performed to detect Reelin-positive neurons. The results indicated a significant decrease in the prefrontal cortex and cerebellar expression of Reelin mRNA, GAD67 protein, and mRNA in BD patients [41].

A three-dimensional study was conducted by G. Rajkowska et al. in 2001 using postmortem brains of 10 BD and 11 control individuals to analyze the same dorsolateral prefrontal area 9 (Brodmann area 9). They observed a reduction in neuronal density in layer III and a diminished number of pyramidal neurons in layers III and IV. Additionally, the sublayer IIIc showed a 19% decrease in glial density, along with morphological changes in glial nuclei [42]. Overall, their study reported a range of alterations in the PFC. 

The amygdala, which holds a strong connection with the PFC, has also been speculated to be involved in neuropathology in BD. Beretta et al., in 2007, measured the size and number of neurons in the amygdala using 12 control subjects and 10 BD patients. They reported a reduction in the number and volume of the lateral nucleus and reduced volume in accessory basal neurons. These findings confirmed the involvement of the amygdala in BD pathogenesis [43].

#### 2.1.2. Genome-Wide Association Studies in BD

In the last two decades, the genetics of BD have been thoroughly researched and significant advances have been made in the field, further highlighting the high heritability nature of this disorder, which is presumed to be 70–90% [44,45]. Genome-wide association studies (GWAS) have enabled the recognition of common genetic variants associated with BD. Baum et al. conducted a GWAS study in 2008. In their research, over 550,000 single nucleotide polymorphisms (SNPs) were genotyped using 461 samples of unrelated BD-I patients and 563 control subjects collected from the National Institute of Mental Health (NIMH). Additionally, they genotyped 772 BD-I and 876 control subjects from a German case sample. One of the identified SNPs strongly associated with BD was in the diacylglycerol kinase (*DGKH*) gene (*p*-value = 1.55 × 10^−8^). This gene encodes for the protein responsible for the lithium-sensitive phosphatidylinositol pathway. Another identified SNP was in the gene *NXN* that encodes the protein nucleoredoxin, which acts as an inhibitor against the Wnt-β-catenin pathway. Three novel proteins, namely, *VGCNL1, DFNB31,* and *SORCS2* were also identified as associated with BD, out of which *VGCNL1* encodes for voltage-gated ion channels [46]. 

In 2011, the Psychiatric GWAS Consortium (PGC) BD Working Group conducted a large-scale GWAS study on BD. They carried out a combined GWAS study of 7481 cases and 9250 control subjects. Cases included patients with BD type I disorder, BD type II disorder, schizoaffective disorder bipolar, and other related bipolar diagnoses. In a replication cohort, they further studied 4496 cases and 42,422 controls and found 34 related SNPs. Eighteen out of 34 SNPs had *p* < 0.05, and 31 out of 34 had SNPs of signals with the same impact direction. The analysis of the combined cohorts consisting of 11,974 BD and 51,792 control cases confirmed *CACNA1C* association with BD and also identified *ODZ4,* which is located in chromosome 11, to be associated with BD [47]. In 2012, Dieset et al. conducted a GWAS analysis using 107 BD type I, 50 BD type II, and 211 healthy individuals. Their analysis reported the involvement of *NOTCH4* along with seven SNPs of the gene region to be associated with increased *NOTCH4* mRNA levels [48]. Lee et al., in 2012, carried out a systematic review of all published GWAS studies collecting literature data from NCBI, Scopus, Web of Science, and ScienceDirect. A total of 14 SNPs and five copy number variations (CNVs) in BD were reviewed, and their report suggested strong associations of *ANK3, CACNA1C, DGKH, PBRM1,* and *NCAN* with BD [49].

In 2015, the Network and Pathway Analysis Subgroup of the PGC conducted a GWAS study using samples collected from 6990 BD and 4820 control subjects. They designed an analysis pathway to rank and identify shared pathways among different disorders (schizophrenia, major depression, and BD). Their results displayed histone methylation processes (*H3K4me3*), which are mainly involved in gene expression and differentiation, as the most significant association and was statistically related to immune and neuronal signaling pathways [50]. In 2016, Chang et al. conducted a GWAS study using 11,564 BD and 17,686 control individuals and found a genetic risk variant rs9836592 in the *CHDH* gene to be significantly associated with BD. In addition, they also reported a uniform upregulation of *CHDH* mRNA expression in the brains of BD patients compared to healthy subjects [51]. 

Forstner et al., in 2017, performed an association test by combining recent schizophrenia GWAS study (35,476 patients and 46,839 controls) with their previous BD GWAS dataset (9747 patients and 14,278 controls). Comparison between the risk alleles of BD GWAS and PGC schizophrenia GWAS revealed that 22 out of 107 schizophrenia SNPs showed an association with BD with a significant *p* value = 1.46 × 10^−8^. *TRANK1* demonstrated a strong association in schizophrenia that has been previously documented as a BD risk gene [52]. Stahl et al. in 2019, carried out a meta-analysis using 20,352 BD and 31,358 control cases. Their results reported 30 loci to be associated with BD. Ten loci were from known genes, whereas 20 loci were novel BD-associated genes (for example, *SCN2A, SLC4A1, GRIN2A, RIMS1*, and *ANK3*). Some of these loci encoded for genes known to regulate ion channels and transporters, neurotransmitter receptors, and synaptic pathways [53].

In 2020, Mullins et al. performed a GWAS study using 41,917 BD and 371,549 control cases. They identified 33 novel genomic loci out of 64 genomic loci that included *CACNB2, KCNB1* genes associated with ion channel regulation in BD. They also reported 17 genomic loci that were previously involved in schizophrenia. Integrating eQTL data, 15 genes (*HTR6, MCHR1, DCLK3, TRANK1*, and *FURIN*) were linked to BD through gene expression [54]. Recently, in 2020, Coleman et al. performed a meta-analysis of data collected from the PGC MD cohort (135,458 cases and 344,901 controls) and BD cohort (20,352 cases and 31,358 controls) with an additional MD cohort from the UK Biobank, which included 185,285 BD cases, 439,741 controls, and non-overlapping 609,424 individuals. Their findings reported 73 genetic loci, out of which 24 new loci were found to be associated with mood disorders. They also calculated a genetic correlation among MD and BD subtypes that showed a stronger correlation of BD type II with MD [55].

#### 2.1.3. Human Cellular Models of BD

Although post mortem experiments can provide evidence regarding structural abnormalities in BD patients, changes in brain function such as cognition, mood, and behavior are highly person-specific, necessitating the need for precision disease modeling. One of the earliest attempts to create a BD model can be traced back to 1997 to a study conducted by Emamghoreishi et al., who established lymphoblast cell lines (LCLs) from 28 BD type I patients, 11 BD type II patients, and 20 healthy subjects as a model to study Ca^2+^ signaling in BD. They measured the intracellular Ca^2+^ concentration using a ratiometric fluorescence assay. Their results showed an increase in the basal Ca^2+^ concentration in the B lymphoblasts of BD type I patients compared to healthy controls, suggesting the existence of irregular calcium homeostasis in the BD patients’ lymphoblasts, especially in BD type I patients [56]. 

Another BD and schizophrenia model using human olfactory neuroepithelial cells obtained from four patients with schizophrenia, four patients with BD, and four control patients was developed in 2013 by Chagoyan et al. They analyzed microtubule organization using neuronal progenitors by immunofluorescence method, then quantified the olfactory marker protein (OMP) using western blot, and performed whole-cell recordings of voltage-activated Ca^2+^ currents by patch-clamp techniques on olfactory sensory neurons obtained from the olfactory neuroepithelium. Their cells were immunostained positive for βIII-tubulin, vimentin, NeuN, and OMP. In contrast, anti-cytokeratin and GFAP labeling showed negative results, confirming the proliferation and differentiation of only neuronal cells. Furthermore, the electrophysiological analysis reported similar L-type Ca^2+^ currents in sensory olfactory neurons obtained from the BD and control groups [57].

Unlike other cellular models, iPSCs have enabled researchers to study viable neurons and glia by providing a rare opportunity to compare gene expression, behavior, drug response, and differentiation to neurons derived from BD patients or healthy controls. In 2014, Chen et al. examined alterations in gene expression patterns of neurons differentiated from iPSCs derived from BD patients. In their study, fibroblast lines from three BD and three control subjects were collected, reprogrammed, and utilized for microarray analysis and calcium signaling. Interestingly, their results showed no difference in the transcriptional profiles of BD and control iPSCs. In contrast, gene expression profiles of neurons isolated from BD iPSCs were substantially different from those of the control iPSCs. BD neurons expressed more membrane receptors and ion channel genes than control neurons [58].

In 2015, Madison et al. investigated the cellular and molecular pathologies of BD by utilizing a familial-based model in which 12 iPSC lines were derived and characterized from two BD-affected brothers and their two unaffected parents. Initially, there were no significant phenotypic variations between iPSCs derived from different family members. However, after neural differentiation, they discovered alteration in the expression of CXCR4 (CXC chemokine receptor-4). CXCR4 in neural progenitor cells (NPCs) from BD patients and their unaffected parents exhibited several phenotypic changes in different stages of neurogenesis and expression of neuroplasticity genes including wnt pathway and ion channel genes [59]. 

### 2.2. Schizophrenia

Schizophrenia is a lifelong mental disorder characterized by varied symptoms that primarily involve a range of cognitive behavior and emotional dysfunctions that can manifest in the form of delusions, hallucinations, disorganized speech, excessive disorganization, catatonic behavior, and negative symptoms including poor community and social functioning [60]. An estimated 21 million people are afflicted worldwide by schizophrenia [61]. Onset of the disease is typically during adolescence, while childhood and late-life onset (over 45 years) are rare.

Men are more likely to be diagnosed with schizophrenia than women. Men also display a worse prognosis than women [62], whereas women tend to be diagnosed later in life than men. For men, the age of onset is between 18 and 25, and for women, it is between 25 and 35, with a second peak occurring around menopause [62,63]. Termed more than a century ago by Dr. Emil Kraepelin as ‘dementia praecox’, schizophrenia was characterized by two main features; first, primarily as a disorder of intellectual functioning compared to manic-depressive illness; and second as an illness with a deteriorating course and a poor prognosis [64]. Subsequently, Eugen Bleuler developed Kraepelin’s ideas and coined the term schizophrenia at the psychiatric community conference in 1908. He identified the basic symptoms as the “four A’s”—ambivalence, autism, affective incongruity and association disturbances—while stating that hallucinations as well as delusions are “secondary symptoms” [65].

Concomitantly, John Hughlings Jackson distinguished the symptoms of schizophrenia as negative and positive symptoms [66]. Positive symptoms mostly include hallucinations, delusions, confused thoughts, decreased spontaneous movement, and disorganized speech. The negative symptoms include the lack of normal human behavior such as alogia (poverty of speech), the flatness of voice, and apathy [64]. The suggested symptoms were later measured in 1991 by Fenton et al. with 187 patients with schizophrenia using the scale for the assessment of positive symptoms (SAPS) and scale for the assessment of negative symptoms (SANS), respectively. The SANS and SAPS ratings of 187 patients suggested that schizophrenia with many negative symptoms was related to poor premorbid, gradual progression, sometimes leading to permanent disability. In contrast, schizophrenia with few symptoms includes good premorbid, acute onset, and better prognosis. However, positive symptoms were moderately less powerful as indicators for disease history, course, or functional disability, and were relatively less effective when predicting future hospitalizations [67].

A study conducted by Peter F. Liddle in 1987 described an association between a group of symptoms, cognitive functioning, and cortical neurological signs in schizophrenia. They performed experiments using 40 patients with schizophrenia within 21–54 years of age. Symptoms were evaluated by three scoring tests: reality distortion syndrome, disorganization syndrome, and psychomotor poverty syndrome. The psychomotor poverty syndrome was associated with evaluating long-term memory, object naming, and conceptual thinking. At the same time, disorganization syndrome assessment was associated with orientation, concentration, and word learning. These two syndromes were linked to a dysfunction in the frontal lobes due to resemblance to two other traditional syndromes, namely pseudodepression and pseudopsychopathic syndrome. Reality distortion syndrome was associated with hallucination, delusion, expressive thought disorder, confusion, and poor figure-ground perception [68,69,70].

#### 2.2.1. Neuroimaging Studies Associated with Schizophrenia

Schizophrenia, which was once considered a psychological disease without any organic brain substrate, is now considered a subject of intensive neuroimaging research. Progress in in-vivo neuroimaging techniques has extensively promoted the study of schizophrenia, linking schizophrenia to abnormal structural and functional connectivity in the brain at various levels [71]. Computerized axial tomography (CT) imaging by Johnstone et al. in 1976 with 17 patients with schizophrenia and eight control subjects reported an abnormal increase in ventricle size in the schizophrenia group, linking to cognitive impairment [72]. Falkai P and Boggarts in 1986 carried out a detailed analysis of cell loss in the hippocampus using postmortem samples of 13 patients with schizophrenia and 11 age-matched control subjects. Their results indicated a distinct reduction in the volume of the total hippocampal region, whole pyramidal band, and the hippocampal segments that include the CA1/CA2, CA3, and CA4 regions. Furthermore, they also observed CA1/CA2, CA3, and CA4 pyramidal cell loss in paranoid patients compared to catatonics [73].

In another study, the total brain volume, brain tissue, and cerebrospinal fluid (CSF) values were measured by Andreasen et al. in 1994 with 52 patients with schizophrenia and 90 control subjects. They reported a reduction in the volume of the total brain tissue of schizophrenia patients. Region-specific abnormalities were primarily observed in the frontal region, whereas the CSF volume was substantially elevated in cortical and cerebellum regions in patients with schizophrenia compared to the controls [74].

A review by Lawrie et al. in 1998 included 40 published MRI studies conducted on 1314 patients (931 male and 383 female) with schizophrenia, and 1172 control subjects (797 male and 375 female) showed a reduction of 3% in the total brain volume in the schizophrenia patients. Specific observations included a decrease in volume of the temporal lobes (6% left, 9.5% right) and amygdala-hippocampal complex (6.5%, 5.5%), while the lateral ventricles exhibited an increase in volume (44%, 36%) in schizophrenia patients. Notably, further analysis indicated that men tended to exhibit a greater loss in volume of amygdala and hippocampal complex [75].

In 2001, Mathalon et al. carried out MRI scans on 24 chronic schizophrenia and 25 control subjects to evaluate whether dysmorphology in schizophrenia patients progressed over time and is associated with the severity of the disease. Their results reported a rapid decrease in the volume of right frontal gray matter and posterior superior temporal gray matter in patients with schizophrenia compared to the control participants. Moreover, the right frontal, left-lateral, and superior bilateral sulci showed increased levels of cerebrospinal fluid [76].

#### 2.2.2. GWAS Studies in Schizophrenia

In the past decade, development in genomic technologies has enabled the identification of genomic associations and vulnerability spots in schizophrenia. In 2008, Donovan et al. carried out screening with Affymetrix GeneChip mapping array for 479 patients with schizophrenia and 2937 control subjects. Their study reported a SNP located in the zinc finger protein *ZNF804A* (*p*-value < 5 × 10^−4^) associated with schizophrenia [77]. Another schizophrenia GWAS study conducted by Potkin et al. in 2009 with 64 schizophrenia and 74 control subjects identified six genes (*ROBO1*-*ROBO2*, *TNIK*, *CTXN3*-*SLC12A2*, *POU3F2*, *TRAF*, and *GPC1*) significantly involved (*p*-value < 10^−6^) in forebrain development and the stress response pathway in schizophrenia [78]. 

In 2011, the Schizophrenia Psychiatric Genomics Consortium (SPGC) published results of a study combining data from the European ancestry cohort (21,856 individuals) and a replication cohort (29,839 individuals) that implicated five new novel loci associated with schizophrenia. Among them, *MIR137*, a micro-RNA, which is a regulator of neuronal development, showed the strongest association in schizophrenia (*p*-value = 1.6 × 10^−11^), and three more loci (*CACNA1C*, *ANK3*, and *ITIH3*-*ITIH4)* were significantly associated with both BD and schizophrenia [79]. 

Multi-stage GWAS analysis conducted by Ripke et al. in 2013, which included 21,246 patients with schizophrenia and 38,072 control cases, identified 22 loci to be significantly associated with schizophrenia. Out of 22, 13 new loci were identified. Five previously identified loci showed significant associations with schizophrenia (*MHC, WBP1L (C10orf26), DPYD-MIR137, SDCCAG8*, and *MMP16*). Three loci have been reported to have combined association with both schizophrenia and BD (*CACNA1C, CACNB2*, and *ITIH3-ITIH4*), and one locus, which was previously found to be associated with BD (*NCAN*) [80].

An extensive GWAS analysis was conducted in 2014 by PGC, which included 36,989 schizophrenia and 113,075 control cases from European and Asian ancestry, which introduced 83 new loci associated with schizophrenia. Some discovered loci were related to glutamatergic neurotransmission (*GRM3*, *GRIN2A, SRR,* and *GRIA1*), and some genes were involved in calcium signaling *(CACNA1C, CACA1I, CACNB2, RIMS1, KCTD13, CNTN4*, and *PAK6*). Interestingly, they found a genome-wide significant association with the *DRD2* gene that encodes for the dopamine D2 receptor. The D2 receptor is widely known as a therapeutic target for antipsychotic medications [81,82,83].

In 2015, Gurung et al. reviewed 40 GWAS studies and addressed the effect of genetic variation on the morphology and function of brain regions in both schizophrenia and healthy controls. They selected 40 studies out of 296 neuroimaging studies based on a quality score, which rated the studies according to supporting evidence. These studies identified four genes (*CACNA1C*, *NRGN*, *TCF4*, and *ZN804A*) involved in gray matter volume. *TCF4* was involved in ventricular volume, *NCAN* and *ZNF804A* were involved in cortical folding and cortical thickness, respectively, and *CACNA1C* and *ZNF804A* were involved in facial recognition [84].

Hou et al., in 2016, performed a meta-analysis on 9784 BD and 30,471 control subjects; *MAD1L1* gene on chromosome 7p22.3 showed a significant association with BD and was previously also reported in BD and schizophrenia [47,85,86]. A transcriptome-wide association study (TWAS) conducted by Gusev et al. in 2018 integrated 79,845 patients with schizophrenia from PGC and 3693 control individuals. They identified 157 TWAS significant genes, 35 of which were not yet recognized as associated loci, and 42 were related to unique characteristics of chromatin remodeling in separate studies [87]. 

In 2018, the BD and schizophrenia working group of PGC identified 32 new loci out of 114 loci from 20,129 BD, 33,426 schizophrenia, and 54,065 control cases that were significantly associated with synaptic and neuronal pathways and were shared between BD and schizophrenia. *DARS2* and *CSE1L* loci were identified to be significantly associated with schizophrenia [88]. Later, Stahl et al. in 2019 identified 30 loci; 20 new loci were considerably associated with BD, eight showed association with schizophrenia, and three loci (NCAN, TRANK1, and chr7q22.3:105 Mb loci) shared associations with both BD and schizophrenia [53]. 

Yu et al., in 2020, performed an integrated study to identify variants that affect methylation and contribute to the risk of schizophrenia. For the analysis, they collected a schizophrenia GWAS dataset of European ancestry from 35,476 schizophrenia and 46,839 control individuals. Furthermore, they performed replication analyses by combining data from the PGC and CLOZUK studies, consisting of 40,675 schizophrenia and 64,643 control cases. Their results indicated 15 SNPs, out of which three risk SNPs (*RERE, ARL6IP4*, and *CENPM*) affected methylation and were suggested to be risk variants that may be involved in the pathogenesis of schizophrenia [89].

Recently, Mahmoudi et al., in 2021, carried out a GWAS analysis using samples from 299 schizophrenia and 131 healthy controls. They found that the *MIR137*4 variable number tandem repeat (VNTR) variant is enriched with a schizophrenia cognitive deficit subtype and was linked to altered brain morphology such as thicker left inferior temporal gyrus and deeper right postcentral sulcus. These results indicated the involvement of *MIR137*4-repeat VNTR in neuroanatomical development, which can also impact the expression of cognitive symptoms in schizophrenia patients [90].

#### 2.2.3. Human Cellular Models of Schizophrenia

Over the years, the number of studies using the schizophrenia cell culture model has risen [91]. Matigian et al., in 2010, developed a neurosphere-derived cell culture model using the patient’s olfactory mucosa. Tissues were collected from nine subjects with schizophrenia and 14 control individuals. They conducted multiple experiments including flow cytometry to characterize the phenotype of six schizophrenia and six control cell lines using several antibodies (CD105, CD73, Oct4, Nestin, TUBB, Sox2, GFAP, and CD45). They also carried out gene and protein expression profiling, qRT-PCR, and western blot. Their results reported high expression of *CD73* and *CD105,* which are known as markers of bone-marrow stromal or stem cells, and low expression of neural progenitor markers. Furthermore, *RELN, RGS4, ERBB3*, and *GSK3* gene expression was downregulated in the olfactory neurosphere-derived (ONS) cells of schizophrenia patients. Using ingenuity pathway analysis, they also determined five main pathways that have been previously identified (Reelin signaling in neurons, VDR/RXR activation, IL-8 signaling, glutathione metabolism, ErbB signaling) to be associated with schizophrenia [92].

In 2011, King et al. used human olfactory epithelium as a model to study alterations in BD and schizophrenia. Nasal mucosa collected from four patients with schizophrenia, four patients with BD, and four control subjects were differentiated into neurons. Using patch-clamp recording, they measured Ca^2+^ currents in olfactory sensory neurons. Their results showed that neural precursors obtained from the nasal mucosa to be an excellent model for studying psychiatric disorders [93].

In 2011, Chiang et al. first isolated iPSCs from a male patient and his sister detected with chronic undifferentiated schizophrenia and chronic paranoid schizophrenia with a *DISC1* mutation using an episomal vector approach. The iPSCs showed morphology similar to human embryonic stem cells and expressed *Nanog, Oct4, Sox2, SSEA3, SSEA4, TRA-1-60, TRA-1-81,* and *TRA-2-49,* which are well-known markers of human iPSCs [94]. During the same year, Brennand investigated the cellular and molecular defects of schizophrenia by reprogramming fibroblasts directly from schizophrenia patients into hiPSCs, concurrently differentiating them into neurons from five patients with schizophrenia and six healthy controls. They performed immunohistochemistry, fluorescence-activated cell sorting (FACS), and neurite analysis, followed by whole-cell patch-clamp recordings. Their results showed that schizophrenia hiPSC-derived neurons exhibited decreased neuronal connectivity combined with a decrease in neurite number, lower postsynaptic density protein (PSD-95) levels, and a reduction in glutamate expression. Additionally, gene expression profiles showed altered expression in cyclic AMP and wnt signaling pathways. Electrophysiology and calcium transient imaging recorded normal inward transient sodium currents and prolonged outward potassium currents. No difference was observed between the spike amplitude of calcium transients in schizophrenia hiPSC neurons and controls [95].

In 2014, Hook et al. investigated the capability of hiPSCs-derived neurons to secrete neurotransmitters in an activity-dependent manner. Fibroblasts were collected from three male patients with schizophrenia, two female control subjects, and one newborn male. Neurons derived from hiPSCs were treated with KCl at a high concentration (50 mM), which resulted in activity-dependent stimulation of neurotransmitter secretion. Radioimmunoassay was performed to measure catecholamines and peptide neurotransmitters. Immunofluorescence imaging confirmed the presence of DOPA decarboxylase, dopamine β-hydroxylase, and phenylethanolamine N-methyltransferase enzymes, which are responsible for the synthesis of dopamine (DA), norepinephrine (NE), and epinephrine (Epi), respectively. Furthermore, the comparison between schizophrenia and control cultures of hiPSC derived neurons reported an elevated high level of secreted DA, NE, and Epi in schizophrenia hiPSC neurons. The cell count of tyrosine hydroxylase (TH) positive cells reported a significant increase in TH positive neurons in schizophrenia hiPSCs neurons compared to healthy controls, suggesting the alteration of the first enzymatic step in the catecholamine biosynthetic pathway [96]

## 3. Mood Stabilizers

### 3.1. Lithium

**Figure 1:**
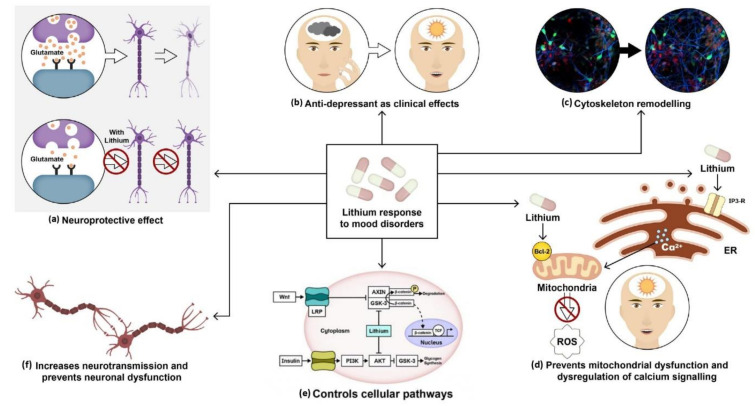
Molecular and therapeutical potential of lithium: (**a**) Lithium prevents neuronal dysfunction by inhibiting the activation of apoptotic pathways. Chronic lithium treatment protects neurons against excitotoxicity. (**b**) Lithium salts have been widely used as anti-manic and anti-depressants. (**c**) It plays a vital role in cytoskeleton remodeling and neurite outgrowth. (**d**) It upregulates neuroprotective protein BCl-2, which prevents mitochondrial dysfunction and calcium channel dysregulation. (**e**) It activates wnt and insulin pathways by inhibiting constitutively activated glycogen synthase kinase-3 (GSk-3) in both pathways. (**f**) It also regulates neurotransmission and prevents synaptic dysfunction.

One of the earliest descriptions of lithium as a treatment for brain disorders in modern medicine can be attributed to Alfred Baring Garrod. He observed uric acid deposition in the blood of patients suffering from gout and suggested using lithium salts in gout treatment including brain gout [1]. A wider usage of lithium in the treatment of mental disorders can be traced back to 1871, when the American physician William Alexander Hammond prescribed lithium salts as the main treatment for acute mania [97]. In 1949, the psychiatrist John F. Cade, utilizing guinea pigs in the study of mental disorders, speculated that conditions such as uric acid toxicity might be a factor that contributes to the symptoms in patients with mania [98]. The anti-manic and prophylactic efficacy of lithium salts were verified in the early 1950s and 1960s through a series of drug trials conducted by Mogens Schou, who strongly confirmed the therapeutic properties of lithium in manic treatment [99]. However, lithium was only approved by the United States Food and Drugs Administration (FDA) for treating mania during the late 1970s after altercations from internal agencies (Figure 1b) [100]. 

Studies on lithium’s mechanism of action have revealed its neuroprotective and anti-apoptotic properties (Figure 1a) [101]. Experiments conducted by Nonaka et al. in 1998 showed that prolonged lithium chloride exposure demonstrated protection of cultured rat cerebellar, cerebral cortical, and hippocampal neurons against glutamate-induced excitotoxicity. The presence of internucleosomal DNA cleavage and chromatin condensation was associated with glutamate-induced delayed neurotoxicity in cerebellar granule cells. Lithium-induced neuroprotection was measured to be therapeutically appropriate at a concentration of around 1.3 mM [102]. 

A study conducted by Hall et al. in 2000 showed that lithium might induce axonal remodeling and neurite outgrowth in several types of cultured neurons (Figure 1c) by mimicking the effect of WNT-7a through the inhibition of glycogen synthase kinase-3β (GSK-3β), thus regulating transcription and indirectly promoting microtubule organization [103]. Other findings by William et al. in 2002 showed a reduction in growth cone collapse by lithium therapy in neuronal cultures using time-lapse video microscopy [104]. Lithium also prevents calcium channel dysregulation and mitochondrial dysfunction, previously reported in various psychiatric disorders (Figure 1d). Schlecker et al. conducted an experiment using PC12 cells to overexpress neuronal calcium sensor-1 (NSC-1), which showed increased intracellular calcium release upon stimulation of the phosphoinositide signaling pathway. This protein family of neuronal calcium sensors is known to regulate Ca^2+^ signaling in neurons [105]. Intracellular calcium was measured in PC12 cells using confocal microscopy. It was observed that lithium treatment on PC12 cells inhibited the effects of NSC-1 [106]. Another study by Chen et al. demonstrated that lithium upregulated mitochondrial Bcl-2 on the mitochondrial outer membrane. Chronic treatment with lithium and VPA revealed increased levels of Bcl-2 in the frontal cortex. Bcl-2 is a neuroprotective protein. It dimerizes with pro-apoptotic proteins and blocks the release of cytochrome C from mitochondria, thus preventing the activation of caspases and programmed cell death (Figure 1d) [107].

Lithium activates both β-catenin and glycogen synthase by inhibiting GSk-3β (Figure 1e) [108]. The inhibition of GSk-3 by lithium affects several pathways as GSK-3 is a kinase responsible for regulating various genes involved in transcription, synaptic plasticity, apoptosis, and cellular structure and function. In the insulin-signaling pathway, the PI3K is activated by insulin binding to the insulin receptor. Activation of PI3k stimulates Akt/protein kinase B, which inactivates GSK-3β by phosphorylation at the serine-rich region and facilitates glycogen synthesis. In the canonical pathway, wnt binds to the frizzled receptor and the low-density lipoprotein receptor-related protein (LRP). This leads to the inhibition of AXIN bound to GSk-3. β-catenin then stabilizes and translocates to the nucleus where it binds to DNA-binding protein to activate the transcription of target genes [109]. 

Several neurotransmitter functions are dependent on G-protein coupled receptors for the activation of phospholipase (PLC) and the generation of inositol 1, 4, 5-triphosphate (IP3), and diacylglycerol (DAG) in the inositol triphosphate pathway. Studies show that lithium can modify neurotransmission by inhibiting inositol monophosphate and inositol polyphosphate-1-phosphatase, resulting in an alteration of the phosphatidylinositol pathways (Figure 1f) [110]. This, in turn, converts ATP into cyclic adenosine monophosphate (cAMP), which leads to modifications in neurotransmitters, hormone release, and changes in tyrosine hydroxylase activity. 

#### 3.1.1. Lithium Treatment in BD Human Studies

In 2010, Lyoo et al. performed longitudinal brain imaging and clinical evaluations of treatment response in 22 patients with BD and 14 control subjects. They analyzed 93 serial MRI images and found that lithium induced a prolonged increase in cerebral gray matter volume. These alterations were linked to lithium’s therapeutic efficacy [111]. In the same year, Benedetti et al. studied the effects of suicidality and lithium on gray matter volumes in BD. They conducted a study on 57 BD subjects with major depressive episodes without psychotic features, 19 with a history of suicide attempts and 38 without any positive history of suicide (controls), 39 lithium-non-treated (11 with positive history of attempted suicide, and 28 with negative history of suicide), and 18 lithium-treated (eight with a positive history of attempted suicide and 10 with negative history of suicide). Voxel-based morphometry was used to evaluate the total and regional gray matter volumes. In the dorsolateral prefrontal cortex, orbitofrontal cortex, anterior cingulate, superior temporal cortex, parieto-occipital cortex, and basal ganglia, they found lower gray matter volumes in BD subjects with suicide attempts. They also found the opposite effect in lithium-treated subjects with increased gray matter volumes in the same areas [112]. In 2011, eleven international research groups provided published and unpublished data about 321 BD and 442 control subjects. The data were used to analyze variations in brain function between patient groups with the help of linear mixed-effects regression models. The analysis showed that BD patients treated with lithium had substantially greater hippocampal and amygdala volumes compared to non-treated patients or healthy control subjects [113].

Preclinical research suggests that lithium may have neurotrophic effects in BD patients’ brains, counteracting pathological processes. In 2002, Bowley et al. conducted an experiment to check abnormalities in the amygdala region using 50-micron post-mortem tissue sections collected from 12 BD and 10 control subjects. Out of 12, 10 BD patient samples were treated with lithium and VPA. Their results showed decreased glial population in lithium or VPA untreated BD patients compared to treated ones [114]. Hibar et al. in 2016 conducted an experiment using postmortem samples from 1710 BD and 2549 healthy controls that showed a reduction in hippocampus and thalamus volume while the lateral ventricles appeared to be considerably larger relative to the controls. They also reported that lithium-treated patients showed substantially greater thalamic volumes than lithium untreated patients [115].

In 2014, Hajek et al. compared manually traced hippocampal volumes using MRI imaging in 37 BD lithium-treated, 19 BD lithium-non-treated, and 50 control subjects. They observed smaller hippocampal volumes in the lithium-non-treated group compared to the control or the lithium-treated groups. These findings support lithium’s neuroprotective properties [116]. In 2015, Gildengers et al. examined cognitive and neuroimaging data in 58 BD and 21 control older adult subjects (~65 years). Subjects underwent extensive neurocognitive assessment and structural brain imaging. Their results showed that long-term lithium treatment was linked to better overall white matter integrity, but not significantly different cognitive function in older adults with BD [117].

Another study in the same year conducted by de Sousa et al. described the connection between oxidative stress in early-stage BD and lithium response. They tested plasma samples from 29 BD subjects treated with lithium for six weeks and 28 healthy control subjects. The analysis was based on the measurement of thiobarbituric acid reactive compounds (TBARS), superoxide dismutase (SOD), catalase (CAT), and glutathione peroxidase (GPx) antioxidant enzyme levels. Their results showed a significant increase in CAT and GPx levels and a decrease in SOD/CAT levels in subjects with baseline BD depression compared to healthy controls. Additionally, TBARS were found to be considerably lower in lithium responders compared to non-responders. These results reinforce the importance of the antioxidant effects of lithium in the prevention of BD progression [118].

In the same year, Findling et al. conducted a study of lithium in pediatric BD. They tested pediatric BD subjects (ages 7–17 years), 53 lithium-treated, and 28 placebo-treated for up to eight weeks. They used the Young Mania Rating Scale (YMRS) score and observed an alteration in the lithium-treated group. The starting dose of lithium was either 600 or 900 mg/day. Subjects weighing less than 30 kg started with 600 mg/day, whereas all other participants began lithium therapy with 900 mg/day. Their results showed that lithium reduced symptoms of mania, was well-tolerated, and was not associated with weight gain, unlike other drugs that are often used to treat youth with BD. These results indicate that the efficacy of lithium in children is similar to that reported in adults [119]. In 2019, Findling et al. studied the role of lithium in the maintenance treatment of youth with BD. They examined pediatric BD subjects (ages 7–17 years) that were treated with lithium for 24 weeks (0.8–1.2 mEq/L). Subsequently, 17 of the responders continued treatment with lithium, and 14 received a placebo for up to 28 weeks. The analysis showed that subjects who continued treatment with lithium had a lower hazard ratio compared to the placebo group, with no evidence for weight gain. These findings support the use of lithium as maintenance therapy in pediatric BD and the safety and tolerability of 28-week lithium treatment [120].

In 2020, Velosa et al. studied the efficacy of lithium related to a lower risk of dementia in BD. The meta-analysis included 10 studies with 6859 BD and 487,966 healthy controls to determine whether BD is a risk factor for dementia. They also compared five studies with 6483 lithium-treated and 43,496 lithium non-treated patients to study the protective effect of lithium in BD. Their results indicate that BD patients are at higher risk of dementia, while treatment with lithium reduces the risk for dementia [121].

#### 3.1.2. Lithium Treatment in Cellular Models of BD 

In 2015, Mertens et al. developed an iPSC BD neuronal model from three lithium responsive (LR) BD patients, three lithium non-responsive (NR) BD patients, and four control individuals to study hippocampal dentate gyrus granule-like neurons of BD patients. Using mitochondrial assays and RNA sequencing, they observed mitochondrial dysfunction in young neurons of BD patients. Furthermore, patch-clamp recordings and somatic Ca^2+^ imaging displayed hyperexcitability of young neurons, which was selectively reversed by lithium treatment only in neurons derived from lithium responding patients [122]. In 2017, Tobe et al. performed protein profiling of hiPSC-derived neurons from seven LR BD patients, three lithium NR BD patients, and seven control individuals to assess the role of lithium in the phosphorylation status of *CRMP2*. Using 2D-differential gel electrophoresis (2D-DIGE) and several pathway analysis (ingenuity pathway analysis and STRING network tool), they observed that the ratio of inactive phosphorylated *CRMP2 (pCRMP2)*: active non-phosphorylated *CRMP2* was elevated in hiPSC-derived neurons from LR BD patients compared to lithium NR BD neurons. *CRMP2*, an intracellular phosphoprotein, plays a pivotal role in dendrite outgrowth, calcium channel regulation, and actin assembly. Immunostaining and western blot analysis showed that lithium significantly reduced phosphorylated *CRMP2* and helps in the remodeling of the cytoskeleton structure, particularly the spine area and density [123]. 

In 2018, Stern et al. used whole-cell patch-clamp recordings to validate the differences in hyperexcitability in the dentate gyrus (DG) granule neurons derived from three LR BD, three lithium NR BD patients, and four control individuals. Unexpectedly, functional analysis carried out by the group indicated varied intrinsic cell parameters in the neurons derived from different subpopulations of the BD patients. They found that elevated fast afterhyperpolarization (AHP) was a distinctive feature of BD DG granule neurons. Fast AHP has been previously implicated in a few types of brain disorders such as Down syndrome [124], epilepsy [125,126], and Parkinson’s disease [127]. In Videlicet, the findings divided BD patients into two subpopulations of neurons according to the patient’s responsiveness to lithium, successfully predicting the patient’s response to the drug solely through electrophysiological recordings [128].

In a follow-up study conducted by Stern al. in 2020, the group expanded their earlier findings using CA3 pyramidal neurons derived from the cohort obtained in the previous study. Their findings showed a hyperexcitability in LR CA3 hippocampal pyramidal neurons compared to control CA3 neurons, though no excitability changes were observed on average in lithium NR CA3 pyramidal neurons. Furthermore, qPCR experiments confirmed overexpression of potassium channels *KCNC1* and *KCNC2* in LR CA3 pyramidal neurons, suggesting that hyperexcitability of BD LR neurons may be related to increased specific potassium currents, enabling sustained firing of neurons. Treatment with lithium reduced the hyperexcitability of LR CA3 neurons by increasing the sodium currents and decreasing fast potassium currents in the LR group [129]. Additionally, several potassium channel blockers also decreased BD neuronal excitability. Therefore, based on their measurements, in 2020, Stern et al. developed a computational model of BD DG granule neurons and CA3 pyramidal neurons that replicated the experimental results. 

A similar phenotype was observed between the simulated neuron and experimental neuron as measured by Stern et al. in 2020 [130]. They also found that lithium non-responsive hippocampal neurons displayed a physiological instability found in the experimental results when reanalyzing the data. This instability caused neurons derived from NR patients to shift their excitability drastically, with minor changes in their sodium currents, resulting in a neuronal network that resided in multiexcitatory states. This instability was caused by a reduction in the sodium current and increased potassium current amplitude. The computational model further confirmed the role of increased potassium currents in BD hippocampal neuronal hyperexcitability [130]. Recently, Mishra et al. (2021) studied lithium effects on circadian rhythms in BD patients’ neurons. A prior study has shown that lithium affects “clock genes” expression and that variations in circadian rhythms can be identified between LR and lithium NR groups [131]. Their study first generated NPCs and glutamatergic neurons derived from iPSCs reprogrammed from five BD and four control donors. They observed strong circadian rhythms in *Per2-luc* expression in NPCs and neurons of LR and controls, but NPC rhythms in LR were shorter whereas NR rhythms were weak and low in amplitude. They found that lithium lengthened the circadian time period in LR and control neurons, but failed to change the rhythms in the lithium NR group [132]. 

#### 3.1.3. Lithium Treatment in Schizophrenia Human Studies

Antipsychotic medications are the primary medication for schizophrenia. However, many patients with schizophrenia do not completely respond to this treatment, and some symptoms persist, even after antipsychotic medication is prescribed. In such cases, lithium acts as an add-on therapy. In 1988, Lerner et al. examined the addition of lithium to haloperidol. They tested 36 patients with schizophrenia and divided them into two groups: 18 patients who suffered from depression and 18 patients that did not suffer from depression. Each group received haloperidol with lithium (300 mg per day) or haloperidol with placebo for eight weeks. Their findings suggest that schizophrenia patients who suffered from depression were the most resistant to neuroleptic treatment and that lithium supplementation was beneficial [133]. 

In 1995, Terao et al. studied the effect of the addition of lithium to neuroleptic treatment in chronic schizophrenia. They examined 21 chronic schizophrenia patients treated with lithium (0.4 mEq/L) or placebo for eight weeks. They found that lithium treatment significantly improved the total Brief Psychiatric Rating Scale (BPRS) scores compared to placebo treatment. However, out of the BPRS subscales, only anxiety–depression levels improved. These findings indicate that adding lithium to neuroleptic therapy reduces anxiety and depression in chronic schizophrenia patients [134]. In 2004, Bender et al. examined the safety and efficacy of lithium treatment combined with clozapine in a sample of 20 patients with schizophrenia. The combined treatment lasted an average of 23.5 months. Lithium was given at a mean maximum dose of 32.4 mmol/d (range 16.2–59.4 mmol/d) and clozapine at a mean maximum dose of 550 mg/d (range 200–1000 mg/d). Their results showed that combined lithium–clozapine treatment can be seen as a safe and successful treatment when used within a moderate dose range and without serotonergic co-medication or other clozapine interfering drugs [135].

In 2006, Kelly et al. tested nine treatment-resistant schizophrenia patients treated with lithium added to clozapine, compared to 25 subjects treated only with clozapine. The mean daily dosage of clozapine monotherapy, adjunct VPA, and adjunct lithium was 506 mg/day, 545 mg/day, and 689 mg/day, respectively. Their results suggested that lithium as an add-on therapy was significantly effective in patients with positive symptoms, anxiety, and depressive symptoms that appeared only during the first month after additional drug medications [136]. Leuct et al. (2015) conducted a meta-analysis of 22 randomized studies using 763 participants. Three studies out of 22 showed that lithium had no advantage over the placebo, and thirteen studies showed that lithium as an adjunct therapy along with antipsychotic drugs had a significant response in participants. However, this effect was irrelevant when schizoaffective disorders were separated [16].

### 3.2. Valproic Acid (VPA)

**Figure 2:**
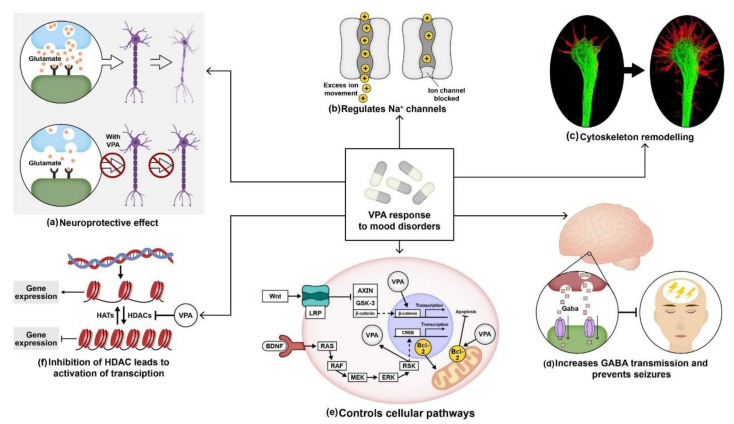
Molecular and therapeutical potential of valproic acid (VPA): (**a**) VPA protects neurons from glutamate-induced excitotoxicity; (**b**) it regulates sodium channels by delaying the activation of voltage-dependent sodium channels; (**c**) like lithium, VPA also helps in cytoskeleton remodeling; (**d**) administration of VPA increases GABA levels and prevents seizures. (**e**) It activates wnt and mitogen-activated protein kinase (MAPK) pathway by blocking constitutively activated glycogen synthase kinase-3 (GSk-3). Furthermore, it also enhances BCl-2 levels that hinder mitochondrial dysfunction. (**f**) VPA also controls the transcription mechanism by inhibiting *HDAC* expression.

VPA, a well-known anticonvulsant drug, is being licensed and used in epileptic seizures and in the treatment of bipolar disorder and other affective disorders. French researcher Pierre Eymard accidentally discovered the anticonvulsant property of VPA. Eymard and two other colleagues, H. Meunier and Y. Meunier, used VPA as a vehicle to dissolve khelline derivatives. They found an anticonvulsant effect of VPA in the pentylenetetrazole seizure test (PTZ) in rabbits [137]. Later in 1995, the FDA approved the antimanic property of this drug [138]. It has been hypothesized that the anti-convulsant property of VPA resembles the therapeutic action of lithium; therefore, it has been prescribed to patients with lithium and carbamazepine resistance [109,139]. VPA has been known to inhibit glutamate excitotoxicity, which leads to neuronal cell death (Figure 2a) [140]. In 2002, Hashimoto et al. reported complete protection of rat cerebral cortical neurons against glutamate-induced excitotoxicity by valproate in a therapeutical dosage-dependent manner. Cerebral cortical cultures were exposed to different glutamate concentrations for 24 h, and it was observed that glutamate-induced cell death with an EC50 at 8–10 µM concentration. Pretreatment of cortical cultures with valproate for six days prevented glutamate-induced excitotoxicity at 1 mM concentration [141]. 

Studies also showed that VPA works by interfering with the regulation of voltage-gated sodium channels (Figure 2b). Van den Berg et al. in 1993 used cultured hippocampal neurons to record fast spatial control of the membrane voltage during measurement of Na+ currents using the whole-cell voltage-clamp method. Alterations in few parameters of Na^+^ current were observed following incubation of the neuronal cultures over 15–30 min in 1 mM VPA. Their results showed that VPA influenced Na^+^ currents in hippocampal neurons, resulting in a substantial reduction in Na^+^ reactivation. Moreover, VPA decreased the peak Na^+^ conductance in a voltage-dependent manner [142]. VPA also increased the growth cone area in cultured sensory neurons (Figure 2c). A study conducted by William et al. in 2002 displayed a reduction in the frequency of collapse and enlargement of growth cones in sensory neurons derived from newborn rat dorsal root ganglia after VPA treatment at a 0.3–0.6 mM concentration [104]. In 1980, W. Loscher et al. reported the therapeutical effect of VPA on inhibitory neurotransmission by collecting blood samples from four volunteers. Their results indicated that subchronic therapy with sodium valproate (VPA) increased the concentration of gamma-aminobutyric acid (GABA) neurotransmitters in the plasma by 30% following two days of oral therapy. On the fourth day, the plasma GABA concentration increased by 90%. Thus, VPA treatment proved to be effective in preventing GABAergic abnormalities. Another study by Frey et al. in 1976 reported the inhibition of seizures induced by picrotoxin using Di-n-propylacetic acid (VPA) in mice and a reduction in neuronal excitability in the brain (Figure 2d) [143,144,145].

VPA was also found to regulate several cellular pathways. Lithium inhibits GSK-3β, causing the stabilization of β-catenin, whereas VPA did not stabilize the β-catenin protein; instead, it increased transcription of β-catenin in the wnt signaling pathway. A study conducted by Wang et al. in 2015 reported increased expression of Wnt-3a and β-catenin in neural stem cells (NSCs) derived from embryonic Sprague–Dawley rats treated with media containing VPA compared to the control group. Using RT-PCR, they showed a higher expression of Wnt-3a, and β-catenin levels in NSCs treated with 0.7 mM VPA compared to non-treated NSCs. Their results suggested VPA induced neuronal differentiation by the activation of the Wnt signaling pathway [146]. As mentioned earlier, chronic treatment of 400 mg/kg/day VPA increased BCL-2 protein levels in the frontal cortex in layer II and layer III in Wistar Kyoto rats, thus inhibiting the activation of caspases and programmed cell death [107].

Recently, VPA has been identified to influence histone deacetylases in the nuclear chromatin structure. Histone deacetylases (HDACs) are chromatin modifiers that are recruited to repress transcription by reducing the degree of acetylation of core histones (Figure 1f) [147]. Phiel et al. in 2001 measured HDAC expression in HeLa cells overexpressing HDAC1 using immunoprecipitation. Their results reported that dose-dependent VPA treatment inhibited HDAC1 expression with IC50 of a 0.4 mM concentration, which is within the therapeutic range of VPA treatment in humans. Furthermore, they also performed immunoblotting using an antibody specific for acetylated histone H4 on Neuro2A cells treated with VPA. Their results showed VPA-induced hyperacetylation of H4 at a concentration as low as 0.5 mM. Histone acetylation is an essential regulatory system for transcriptional regulation. Non-histone proteins such as P53 were also hyperacetylated by VPA at a concentration as low as 1–2 mM [148]. 

#### 3.2.1. VPA Treatment in BD Human Studies

The increase in the percentage of poor or non-responders as well as patients intolerant to the side-effects of lithium has paved the way toward identifying new alternative treatment options. Therefore, anticonvulsant drugs have been studied as a possible mood stabilizer since the early 1970s [149]. In 1988, Mcelroy et al. conducted a study on six patients with rapid-cycling BD. Earlier reports of the patients showed poor response to lithium and carbamazepine. However, VPA treatment at a concentration greater than 50 mg/L showed a positive response within one to two weeks. They observed improvement in symptoms of mania and tricyclic-induced mania with no signs of depression [150]. Similarly, Calabrese et al., in 1992, examined VPA’s spectrum of efficacy in 78 patients with rapid-cycling BD in a 15.8-month trial. There were 30 patients who received VPA monotherapy and 48 received a combination therapy. The treatment procedure was non-randomized and related to prior treatment history (VPA dosage of 1498 mg/day). Their treatments showed an acute response in 54% of the patients with mania, 87% of mixed states patients, and 19% of patients with depression. They also observed prophylactic responses in 72% of the patients with mania, 94% of mixed-state patients, and 33% of patients with depression [151].

In 1997, Denicoff et al. carried out a clinical trial to check the efficacy of VPA plus lithium and triple therapy with lithium, carbamazepine, and VPA in 24 BD patients. Out of 18 patients evaluated, six had mild to strong VPA plus lithium response, four out of six did not respond to any previous treatment, and three out of seven patients responded to triple therapy. Their findings showed that BD patients resistant to lithium or carbamazepine showed significant prophylactic benefits when treated with lithium plus VPA or triple therapy [152]. Schneider et al. in 1998 reported in a case series of four elderly patients (mean age 74–75 years) with rapid cycling BD. VPA (750–1400 mg/day) along with lithium was administered to these patients. The report described that VPA might enhance lithium sensitivity within the population and potentially results in lower lithium concentration therapy needed with a greater degree of protection [153]. In 2001, Bowden et al. compared the efficacy of VPA, lithium, and the placebo as prophylactic therapy. Randomized maintenance therapy was carried out with VPA, lithium, or placebo in a 2:1:1 ratio in 372 BD patients who met the recovery criteria within three months of the onset of manic episodes. Their findings showed that for a chronic mood episode or a depressive episode, VPA was more effective than the placebo. Furthermore, in a longer duration active prophylaxis study, VPA was superior to lithium and had less deterioration in depressive symptoms and global assessment scale scores [154].

Another study was conducted by Salloum et al. in 2005 to examine the effectiveness of VPA in reducing alcohol consumption and stabilizing mood symptoms in BD patients. Fifty-nine patients with BD and alcohol dependence participated in the trial. They received a VPA dosage of 750 mg/day, which was further increased to reach a serum concentration of 50 to 100 µg/mL. They observed that the VPA group had significantly fewer drinks per heavy drinking day. Higher VPA serum concentrations were found to be associated with better alcohol tolerance. Furthermore, symptoms of mania and depression also showed improvement with the VPA treatment [155]. Geddes et al. in 2010 tested 330 patients with BD-I to evaluate whether lithium and VPA together were more effective than either drug alone in preventing BD. Patients were randomly assigned to 0.4–1.0 mM of lithium monotherapy (n = 110 patients), 750–1250 mg of valproate monotherapy (n = 110 patients), and a combination of both drugs for 4–8 weeks (n = 110 patients). Their results indicated that a combination of lithium and VPA therapy was more likely to prevent relapse than VPA monotherapy [156].

In parallel, Smith et al. in 2010 performed a meta-analysis study of trials carried out in acute BD patients. Four randomized control trials with 142 participants were included. The duration of the studies was six weeks for two studies and eight weeks for the other two studies. VPA doses were given at 250 mg and diluted up to mean serum levels between 61.5 and 83 µg/mL. Their results reported a substantial decrease in depression with 50% of the participants that displayed improvement in depression [157]. In 2011, Meltzer et al. studied the adjunctive effect of VPA along with olanzapine and risperidone using 160 patients with DSM-IV-TR-defined schizophrenia, schizoaffective disorder, or BD after 1, 3, 6, and 12 months of treatment. Changes were observed in triglycerides (TG) and TG/high-density lipoprotein cholesterol (HDL-C) ratio, both of which are risk factors for ischemic cardiovascular disease. Glycosylated hemoglobin (HgbA1c), body mass index (BMI) were the key factors for the study. Their results displayed that at three and six months, olanzapine plus VPA produced a significantly greater increase in HgbA1c, BMI, weight, TG, and TG/HDL-C than olanzapine/-VPA, while risperidone/+VPA produced substantially smaller increases in HgbA1c, BMI, weight, TG, and TG/HDL-C than the risperidone/-VPA group. Thus, VPA may have a wide range of metabolic effects when combined with different antipsychotic drugs [158].

Another study carried out by Chen et al. in 2014 investigated the efficacy of VPA as a monotherapy or with dextromethorphan (DM) and observed an alteration of inflammatory cytokines and neurotrophic system dysfunction in BD. A total of 309 BD and 123 healthy controls were randomly allocated to one of the three groups: VPA (30 or 60 mg/day) plus DM30 mg/day, VPA plus DM60 mg/day, or VPA plus placebo for 12 weeks. Their results showed higher plasma cytokine and lower plasma BDNF levels in BD patients before treatment. However, after treatment, plasma cytokine levels reduced for all the groups, and plasma BDNF levels increased significantly in the VPA plus DM60 group. They concluded that VPA combined with dextromethorphan substantially improved the neurotrophic activity in BD patients than the VPA treatment alone [159].

#### 3.2.2. VPA Treatment in Cellular Models of BD

Previous GWAS studies have already identified the *TRANK1* gene loci to be significantly associated with BD [85,160,161]. A study conducted by Jiang et al. in 2019 showed that VPA retrieves the expression of *TRANK1* in iPSC-derived neural cells. Prior studies by the same group reported that VPA increased the expression of *TRANK1* from shallow baseline levels in immortalized, non-neural cell lines [160]. In this study, they derived neurons and astrocytes from iPSCs generated from fibroblasts of two BD patients, one spinomuscular atrophy I patient, and eight healthy controls. They performed gene expression profiles for iPSCs, NPCs, and neurons that were validated with qRT-PCR analysis. Their results showed that VPA treatment increased *TRANK1* expression in iPSCs and NPCs from 3- to 6-fold after 72 h of VPA treatment, whereas VPA treatment in astrocytes and neurons showed no significant change, indicating the cell type-specific mechanism of action of VPA in actively dividing cells compared to differentiated ones. They also compared *HDAC1* and *TRANK1* expression in iPSCs, NPCs, neurons, and astrocytes. Their results showed increased *TRANK1* expression over baseline in neurons whereas *HDAC1* expression was highest in iPSCs and NPCs. Based on their results, they suggested a comparable hypothesis in which *TRANK1* is stabilized by *HDAC1* inhibition caused by VPA [162]. 

Recently, Santos et al. studied iPSC-derived DG granule neuron transcriptome from BD patients and healthy individuals. Their cohort consisted of three LR BD, three NR BD, and four healthy controls. Transcriptomic profiling of DG neurons derived from LR and NR BD patients showed that the Wnt/β-catenin signaling pathway was impaired in NR neurons with a substantial reduction in the expression of *LEF1* transcription factor. The treatment of VPA increased *LEF1* and the activity of β-catenin expression. Moreover, VPA treatment also reduced the hyperexcitability in NR neurons [163]. 

#### 3.2.3. VPA Treatment in Schizophrenia Human Studies

VPA has been proposed for the treatment of psychiatric or cognitive symptoms as an alternative in schizophrenia. Early clinical efficacy of VPA has been already reported, particularly in schizophrenia patients with acute illness. In 1999, Facciola carried out two studies to evaluate the effect of VPA in schizophrenia patients that were also treated with clozapine on the steady-state clozapine plasma levels as well as on its key metabolites norclozapine and clozapine N-oxide. First, they compared the concentration of clozapine and its metabolites between 15 patients administered with VPA combined with clozapine and 22 patients treated only with clozapine. They observed higher levels of clozapine and lower levels of norclozapine in patients treated with VPA compared to clozapine alone. In a follow-up study, they measured plasma concentrations of clozapine in six schizophrenia patients before and after VPA treatment (900–1200 mg/day) for four weeks. No substantial change was observed during the study. However, a tendency was reported for higher clozapine levels and less norclozapine after VPA treatment [164]. Wassef et al. in 2000 examined the effect of VPA as an add-on treatment to haloperidol using 12 hospitalized patients with chronic schizophrenia. During the clinical trial, patients received 10 mg of haloperidol for three days, followed by 15 mg for the next 18 days. Furthermore, 75 g/mL of VPA were administered to five patients for two weeks, and the other seven were given a placebo. Their results displayed that on day 21, the VPA group scored higher on the clinical global impression scale (*p* ≤ 0.04), BPRS (*p* ≤ 0.13), and schedule for assessment of negative symptoms scores (*p* ≤ 0.007). Thus, they concluded that the addition of VPA to standard antipsychotic drugs might help alleviate the symptoms of schizophrenia [165].

Another study by the same group in 2001 carried out a comparative study using 30 patients who received standard antipsychotics combined with VPA during their clinical therapy compared to those who did not receive any augmentation. In their study, patients hospitalized for an acute exacerbation of schizophrenia received either VPA augmentation (early-augmentation group) or started receiving haloperidol solely three days after starting treatment (non-augmentation group). The non-augmentation group did not respond to haloperidol for 14 days and was later administered with 20 mg/day of VPA. The treatment was scaled based on factors such as suspiciousness, hallucinations, unusual thought content, and emotional withdrawal. The results demonstrated a 32.4% improvement in the early augmentation group compared to the non-augmentation group. Moreover, 50% of the patients in the non-augmentation group did not respond to haloperidol alone for two weeks. However, VPA augmentation caused an improvement of 29% [166].

In 2000, Citrome et al. carried out a survey describing changes in the additional use of VPA and other mood stabilizers among schizophrenia patients from 1994 to 1998. Their analysis reported that the adjunctive use of VPA tripled from 1994 to 1998 in schizophrenia patients. In 1994, lithium was the most commonly prescribed mood stabilizer for 13.2% of the patients followed by VPA. Whereas, in 1998, 35% of patients were prescribed with VPA treatment (1520 mg/day), followed by lithium [17]. In 2003, Casey et al. evaluated the adjunctive property of VPA along with other antipsychotic agents in 249 schizophrenia patients. In their study, the patients were randomly allocated to olanzapine monotherapy, risperidone monotherapy, VPA (15 mg/kg/day) plus olanzapine, or VPA plus risperidone for 28 days. Their results showed baseline improvements, which were significantly higher in the combination therapy within three days compared to monotherapy [167]. In a retrospective study conducted by Kelly et al. in 2006, VPA or lithium was added to clozapine in treatment-resistant schizophrenia patients and was compared with clozapine monotherapy. Both treatment groups showed comparable increases in the total BPRS scores after six months. However, patients on VPA or lithium demonstrated slightly greater improvement in the first month than those on clozapine alone. Clozapine monotherapy resulted in an average weight gain of 8.7 pounds, 3.0 pounds with VPA, and 13.3 pounds with lithium. Furthermore, adjunct treatment with VPA in the first month was also effective in reducing aggression and anxiety-like symptoms [136].

In 2008, Sajatovic et al. conducted a 12 week open-label study to evaluate the effect of adjunctive anticonvulsant medication in 20 older schizophrenia patients (mean age of 61 years). The average dose was 587.50 mg/day for the extensive release of VPA administered along with other antipsychotic treatments. Their outcomes showed psychosis scores calculated by positive and negative syndrome scale (PANSS), global functioning as tested by global assessment scale (GAS), and depression, as measured by geriatric depression scale (GDS), to be significantly reduced in the patients. Thus, they concluded that an extended-release of VPA in older schizophrenia patients appeared to be effective and well-tolerated [168]. 

## 4. Limitations in the Use of iPSCs for Modeling Psychiatric Disorders

Although these new technologies have enabled us to rapidly improve our understanding of psychiatric disorders, a barrier still exists between our existing information and the effective treatments for patients. In light of the variability of the symptoms and genetic factors associated with a psychiatric disorder, the limitation associated with iPSC studies is the difficulty of collecting and maintaining large cohorts of iPSC lines due to the high costs of reprogramming and differentiation factors. This disadvantage renders with the study of common variants associated with a particular psychiatric disorder and limits the use of these models for common drug design. The genetic complexity of most psychiatric disorders makes the variability broader and small cohorts may not be suitable enough to find the common phenotypes and how to treat them [169]. Another constraint is that these models differ in several aspects from in vivo brain development. The iPSC’s neuron generation protocols are not fully developed and the resulting neuronal population is still not well characterized. As a result, many hiPSC-derived neurons are immature, reaching only prenatal stages with immature ion channels and synaptic connections. This was proven by the fact that only a small portion of the cells fire mature action potentials [170,171]. Furthermore, protocols do not exist for many neuronal types and sub-types and glia in the culture dishes are often lacking or cannot reach full physiological development. As with both cells and organoid systems, most models can be cultured for a short time and are mostly utilized to investigate early embryonic brain development. One evident concern is the detachment of cells after three months post-seeding in the neuronal cultures. For organoids that can reach later stages, there is cell necrosis in the center of the organoids [172,173]. Furthermore, it is unclear how far the cells within organoids can acquire or mimic the structural and physiological features of the postnatal brain. Various mental disorders exhibit defects in later stages of development and postnatal periods. These critical phases are not properly recapitulated within organoids or culture systems as most of the protocols are involved in the mid-gestational stages of brain development, preventing the investigation of synaptic and circuit-level dysfunction during child development or at later stages [174]. The organoids system currently lacks many cell types and is composed mainly of neurons. Like any other model system, it is vital to understand the limitations of the iPSC system to draw significant conclusions for future research.

## 5. Conclusions

Psychiatric conditions continue to be a global mental health problem, affecting a growing number of people each year. The prognosis of these diseases is influenced by genetic, epigenetic, and environmental factors. Two of the most common mental illnesses are BD and schizophrenia. Recent advances in neuroimaging, genetics, epigenetics, and cellular modeling have advanced our knowledge about the underlying processes of these psychiatric disorders. In BD, advances in neuroimaging using MRI and CT scans have provided valuable knowledge about structural anomalies in the hippocampus, thalamus, cortex, and amygdala. Structural changes in schizophrenia include a reduction in the lateral ventricle, hippocampus, and frontal cortex. GWAS analysis has also found many common genetic associations, many of which confer significant risk for disease. Several of these genomic markers are shared between BD and schizophrenia such as *CACNA1C, CACNB2, NCAN, TRANK1, ITIH3-ITIH4*, and *ANK3.*

Mood stabilizers are often prescribed for the treatment of psychiatric patients. For decades, lithium has become the gold standard in BD treatment due to its effective treatment in BD patients with mania and depressive symptoms. Studies conducted with BD patients have shown that lithium successfully reduces the severity and frequency of mania and relieves bipolar depression. In schizophrenia, lithium in combination with neuroleptic drugs has also shown efficacy on psychotic symptoms in a certain percentage of patients. VPA, on the other hand, has been successful in treating acute mania in BD. Studies show that VPA can be used in BD patients with rapid-cycling and mixed or dysphoric mania as a monotherapy or as an adjunct to lithium or carbamazepine. Moreover, VPA has also been used as an add-on therapy for schizophrenia in a few comparative trials alongside other antipsychotic drugs.

The generation of iPSCs has provided various human-derived cells, allowing better modeling of psychiatric disorders and tailoring patient-specific investigation. In BD, patient-derived neurons displayed many neuronal anomalies including excitotoxicity, elevated mitochondrial activity, calcium channel dysregulation, and hyperexcitability in hippocampal neurons. Interestingly, these aberrations occurring in iPSC-derived neurons were only reduced by lithium treatment in neurons derived from LR BD patients (but not in NR BD patients). Treatment with VPA upregulated *LEF1* expression, accelerated transcriptional activity of β-catenin/*TCF/LEF1* in NR neurons, and reduced the hyperexcitability in both LR and NR BD neurons. In schizophrenia, the use of iPSC models to analyze disease pathologies has also aided in investigating several molecular etiologies, which include altered differentiation of patient-derived neural stem cells, abnormal NPC proliferation and migration, decreased neuronal integration and neurite number, impaired synaptic activity, increased protein translation, and dysfunctional gene expression patterns in different signaling pathways. Thus far, the action of lithium and VPA mechanisms in BD remain to be further deciphered, and even more so in schizophrenia. Further research using cellular models and iPSCs is required to elucidate the pathophysiology of these psychiatric disorders and the action by which mood stabilizers affect the patients’ brains.

## Data Availability

Not applicable.
